# Comparison of the efficacy and safety of different puncture routes for ultrasound-guided fascia iliaca compartment block for early analgesia after hip arthroplasty: A meta-analysis

**DOI:** 10.1097/MD.0000000000039313

**Published:** 2024-08-30

**Authors:** Zhi Yang, Wang Xu, Shan Xu

**Affiliations:** a Department of Anesthesiology, The First People’s Hospital of Yongkang, Zhejiang Province, China; b Department of Anesthesiology, Orthopaedic Hospital of Yongkang, Zhejiang Province, China.

**Keywords:** analgesia, fascia iliaca compartment block, hip arthroplasty, meta-analysis, routes

## Abstract

**Background:**

This study aimed to compare the effect of ultrasound-guided fascia iliaca compartment block with different puncture sites on postoperative analgesia in patients undergoing hip arthroplasty.

**Methods:**

We searched the PubMed, Web of Science, EBSCO, Wiley Library, Embase, China National Knowledge Infrastructure, and Wanfang databases for literature on ultrasound-guided fascia iliaca compartment block through different puncture sites in hip replacement patients. The software package R (4.2.1) was used in the meta-analysis.

**Results:**

The meta results showed the suprainguinal approach (SA) puncture pathway had a significantly longer operative time than the infrainguinal approach (IA) pathway (mean deviation [MD] = 0.97, 95% confidence interval [CI] [0.09, 1.84], *P* < .01) when performing orthopedic surgery. In terms of nerve block efficacy, only the block rates of the obturator nerve, lateral femoral cutaneous nerve, and femoral nerve groups did not show significant differences between the SA and IA pathways. In contrast, the results of the Meta combined effect size of visual analogue scale scores during postoperative activity showed that the SA puncture pathway significantly reduced patients’ pain scores compared with the IA pathway at the T1 (3–6 h) and T2 (8 h) time points (MD = −0.39, 95% CI [−0.77, −0.01], *P* = .04 in the T1 group; MD = −0.58, 95% CI [−0.95, −0.21], *P* < .01). The differences in pain scores at the T3 (12 h) and T4 (24 h) time points were not significant, and in terms of adverse reaction rates, the differences in the incidence of pruritus, sedation, urinary retention, and nausea and vomiting were not significant.

**Conclusions:**

This study demonstrates that the SA puncture pathway has a significant advantage over the IA pathway in reducing active pain in early postoperative pain management without increasing the risk of adverse events. This finding supports the prioritization of SA pathway in clinical practice where postoperative pain control is considered. Future research should continue to explore the use of SA pathway in different patient populations and types of surgery, as well as their impact on long-term postoperative recovery, with the aim of optimizing individualized postoperative pain management strategies.

## 1. Introduction

Hip fracture is one of the most common injuries caused by senile osteoporosis, accounting for more than 20% of all fractures in the elderly. With the accelerating trend of population aging, the incidence of hip fractures continues to increase every year, reducing the quality of life of patients.^[1,2]^ In this background, hip resurfacing has become the main surgical option for the treatment of end-stage hip pathology and its application is gradually expanding.^[3,4]^ A total of 572,000 hip replacements are expected to occur in the United States by 2030, and it is estimated that there will be more than 6.26 million hip-fracture patients by 2050.^[5]^ Despite the critical importance of hip arthroplasty for the treatment of femoral fractures, postoperative pain management remains a major clinical challenge. During hospital stay, patients were managed with a comprehensive approach that included pain control measures, early mobilization, and multidisciplinary assessments involving physiotherapy and occupational therapy, which aimed to enhance recovery and minimize functional decline. Additionally, regular evaluations of activities of daily living (ADL) were conducted to monitor progress and adapt care plans accordingly.^[6,7]^ Effective pain control strategies, aimed at reducing the patient’s postoperative pain while reducing dependence on pain medication and the adverse effects it may cause, have become a key element of modern hip reconstruction surgery.^[8,9]^ Among the many pain management methods, the fascia iliaca compartment block (FICB) is a popular regional anesthesia technique in orthopedic surgery, which is mainly used in lower limb surgery, where regional nerve blocks are performed by subcutaneous injection of medication.^[10,11]^ FICB has been shown to reduce opioid consumption and lower pain scores after hip arthroplasty and is one of the main methods for pain control after nerve block in hip arthroplasty patients.^[12]^

An ultrasound-guided suprainguinal approach (SA) has recently been proposed as a new method for FICB. Compared with the classic infrainguinal approach (IA), SA facilitates diffusion and improves the analgesic effect when small amounts of drugs are used.^[13,14]^ However, in clinical application, controversy remains over the analgesic effect of the IA and complication rate of different puncture sites for FICB.^[12]^ Controversy has centered on several areas, firstly although both SA and IA are effective in providing postoperative pain control, there is no conclusive evidence as to which method provides longer lasting and more effective pain relief.^[15–17]^ Secondly, the differences between the 2 methods in terms of drug diffusion efficiency, range of nerve block, and effectiveness of the block on specific nerves (e.g., the obturator nerve) are crucial in determining the optimal clinical application.^[18,19]^ In addition, the comparison of SA and IA methods in terms of complication rates (e.g., local hematoma, infection, and side effects due to systemic absorption of drugs) is a factor that must be taken into account when selecting the optimal puncture technique.^[13]^

Therefore, the aim of this study was to comprehensively assess the existing studies on the analgesic efficacy and safety of the 2 FICB puncture routes, SA and IA, in postoperative total hip arthroplasty patients by means of a systematic literature review and meta-analysis. By comparing the difference in effectiveness between the 2 techniques, we can provide a more scientific basis for the selection of FICB methods in clinical practice. In particular, the potential differences between the 2 methods in terms of ease of operation, pain control effectiveness, extent of drug diffusion, and risk of complications were considered. The aim of this meta-analysis is to reveal which method has a significant advantage in providing optimal pain management after total hip arthroplasty in order to optimize postoperative pain management strategies and improve patient comfort and satisfaction after surgery.

## 2. Methods

### 2.1. Literature search strategy

This meta-analysis followed PRISMA guidelines. We searched the PubMed, Web of Science, EBSCO, Wiley Library, Embase, China National Knowledge Infrastructure, and WanFang databases for publications. Our search for studies was restricted to English or Chinese-language journal articles published from the database’s inception date until December 17, 2023. English-language databases were searched using the retrieval words “Hip joint replacement,” “Vertical inguinal approach/horizontal inguinal approach,” “Suprainguinal/Infrainguinal,” “Ultrasound,” “analgesia,” “Fascia iliac space block,” “Hip Arthroplasty”, and “FICB.” The search terms in the Chinese databases were the translation of the above words.

Journal articles that fulfilled the following criteria were included: (i) patients of all ages with hip arthroplasty (total or half hip); (ii) participants received ultrasound-guided fascia iliaca block; (iii) the types of studies were randomized controlled trials (RCTs); and (iv) the outcome metrics consisted of 1 or more of the primary outcome metrics of time to puncture, visual analogue scale (VAS) score at the time of activity, nerve block rate, and secondary outcome metrics of adverse events. The exclusion criteria were as follows: (i) subjects did not undergo hip replacement; (ii) repeated published literature or non-RCT studies; (iii) the full-text could not be obtained or the study data could not be extracted; and (iv) abstracts, reports, reviews, or conference literature.

### 2.2. Literature quality evaluation

Two researchers independently conducted the literature search according to agreed English and Chinese retrieval words and screened the literature based on the established inclusion and exclusion criteria by reading the titles, abstracts, and full texts. Disagreements between the 2 researchers or discrepancies in the literature evaluation were resolved by a third researcher. Literature quality evaluation was performed based on the Cochrane Handbook for Systematic Reviews of Interventions and included evaluation of selection bias (random sequence generation and allocation concealment), performance bias (double blinding), measurement bias, follow-up bias, and reporting bias.

### 2.3. Data extraction

Extracted information included the authors (first author or the co-first author), year of publication, characteristics of the subjects [number of cases in the 2 groups, age, sex ratio, and body mass index], outcome indexes [procedure duration, nerve block rate (femoral nerve/lateral femoral cutaneous nerve/obturator nerve), VAS score during activity, and incidence of adverse reactions (pruritus/nausea/vomiting/sedation/urine retention)].

### 2.4. Statistical analysis

The statistical analysis was performed by the authors of the study. R 4.2.1 software was used for meta-analysis. The ratio of relative risk (RR) was used as the effect size for dichotomous data, such as the nerve block rate and the incidence of adverse reactions. The mean difference (MD) was the effect size for continuous variable data, such as the procedure time and VAS score. Heterogeneity among the included studies was evaluated using Cochran Q and I^2^ statistics. I^2^ ≥ 50% indicated significant heterogeneity among the included studies, with pooled estimates calculated using a random-effects model in the meta-analysis. Subsequently, subgroup and sensitivity analyses were conducted to explore the sources of heterogeneity and test the stability of the results. When I^2^ < 50%, indicating low heterogeneity, a fixed-effect model was used for meta-analysis.

## 3. Results

### 3.1. Literature search process

**Figure 1.**
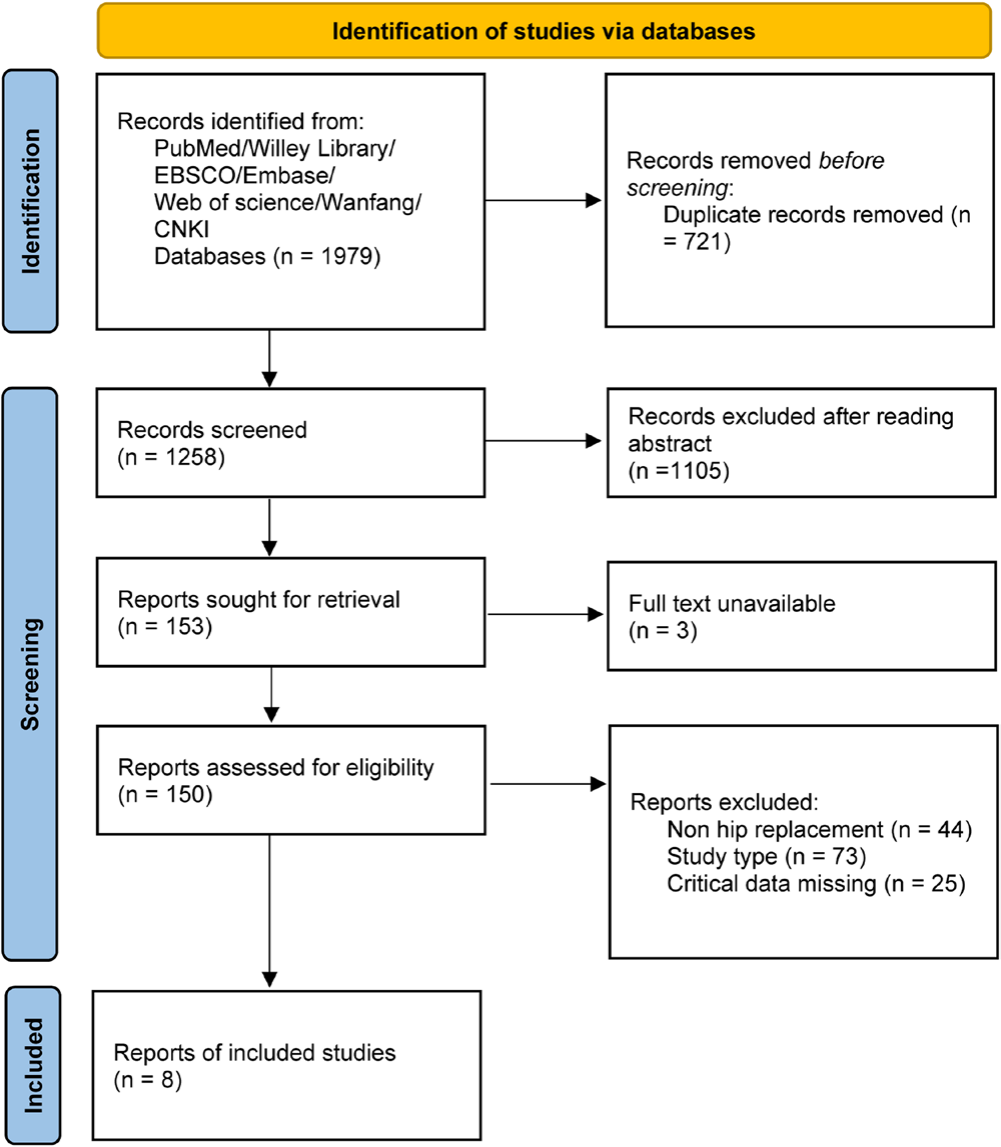
Flow chart of the literature search.

We identified 1979 records by searching PubMed (n = 854), Wiley Library (n = 63), Web of Science (n = 174), EBSCO (n = 221), Embase (n = 396), WanFang (n = 5), and China National Knowledge Infrastructure (n = 35). After removing duplicates, we screened 1258 records, and after removing irrelevant studies, 153 records remained. Three records could not be obtained in full-text. After reading the entire 150 articles obtained, a secondary screening was conducted, and the remaining 8 articles were included in the study. A total of 452 subjects, including 228 patients in the SA group and 224 in the IA group, were included in the analysis. The literature screening process and results are shown in Figure 1. The distribution of SA and IA groups in the included literature was similar in terms of sex ratio (male/female), age, and body mass index, and the number of enrolled cases varied slightly between studies, ranging from 18 to 40 in the SA group and 20 to 38 in the IA group, with the age span ranging from 41.0 to 73.8 years, as detailed in Table 1.

**Table 1:** Characteristics of studies included in the meta-analysis.

Study	Study design	Participants		Gender (males/females)		Age (years)		BMI		Outcome
		SA	IA	SA	IA	SA	IA	SA	IA	
Chen 2021^[11]^	RCT	18	20	6/12	6/14	72.1 ± 6.0	73.8 ± 5.4	22.4 ± 4.1	21.5 ± 3.6	①②③
Fan 2019^[18]^	RCT	30	26	18/12	12/14	66.9 ± 13.0	64.7 ± 10.8	–	–	①②
Feng 2022^[17]^	RCT	30	30	14/16	15/15	59.3 ± 7.8	60.2 ± 9.4	24.5 ± 0.58	24.8 ± 0.74	①
Kumar 2015^[13]^	RCT	20	20	14/6	11/9	41.0 ± 15.4	45.3 ± 12.8	23.3 ± 3.3	24.5 ± 3.3	③④
Pu 2021^[14]^	RCT	40	38	22/18	23/15	69.72 ± 5.64	68.94 ± 6.33	23.16 ± 4.39	23.31 ± 3.78	③④
Wang 2015^[15]^	RCT	30	30	14/16	13/17	62 ± 10	60 ± 10	26 ± 6	25 ± 6	②④
Yu 2019^[16]^	RCT	30	30	14/16	15/15	71 ± 6	71 ± 5	23 ± 5	23 ± 4	①②
Zhang 2016^[19]^	RCT	30	30	14/16	13/17	70.20 ± 4.04	69.29 ± 4.33	24.84 ± 2.12	24.93 ± 2.39	①②③④

BMI = body mass index, IA = infrainguinal approach, RCT = randomized controlled trial, SA = suprainguinal approach. Outcome indicators: ① procedure time; ② nerve block rate (lateral femoral cutaneous nerve/femoral nerve/obturator nerve); ③ VAS score during activity, T1: 3 to 6 hours, T2: 8 hours, T3: 12 hours, T4: 24 hours; ④ incidence of adverse reactions (pruritus/nausea and vomiting/sedation/urine retention).

### 3.2. Quality evaluation of selected literature

**Figure 2.**
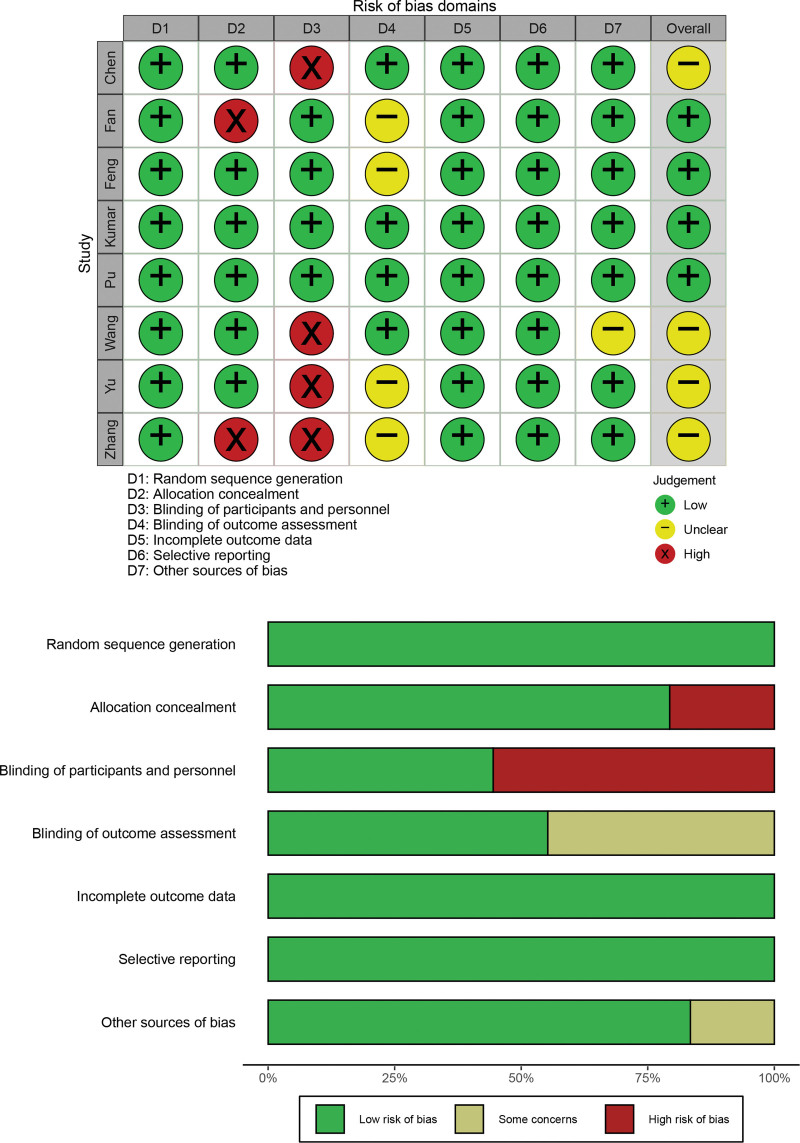
Evaluation of the risk of bias of the included studies.

The included studies were evaluated for risk of bias based on the Cochrane Handbook for Systematic Reviews of Interventions, as shown in Figure 2. The literature quality evaluation found that among the 8 included studies, 4 studies had partial indicators of “unclear risk of bias” or “high risk of bias,” while the remaining 4 studies had all indicators of “low risk of bias,” which met the requirements for meta-analysis of literature risk bias.

### 3.3. Effect of SA and IA puncture routes on nerve block rates

**Figure 3.**
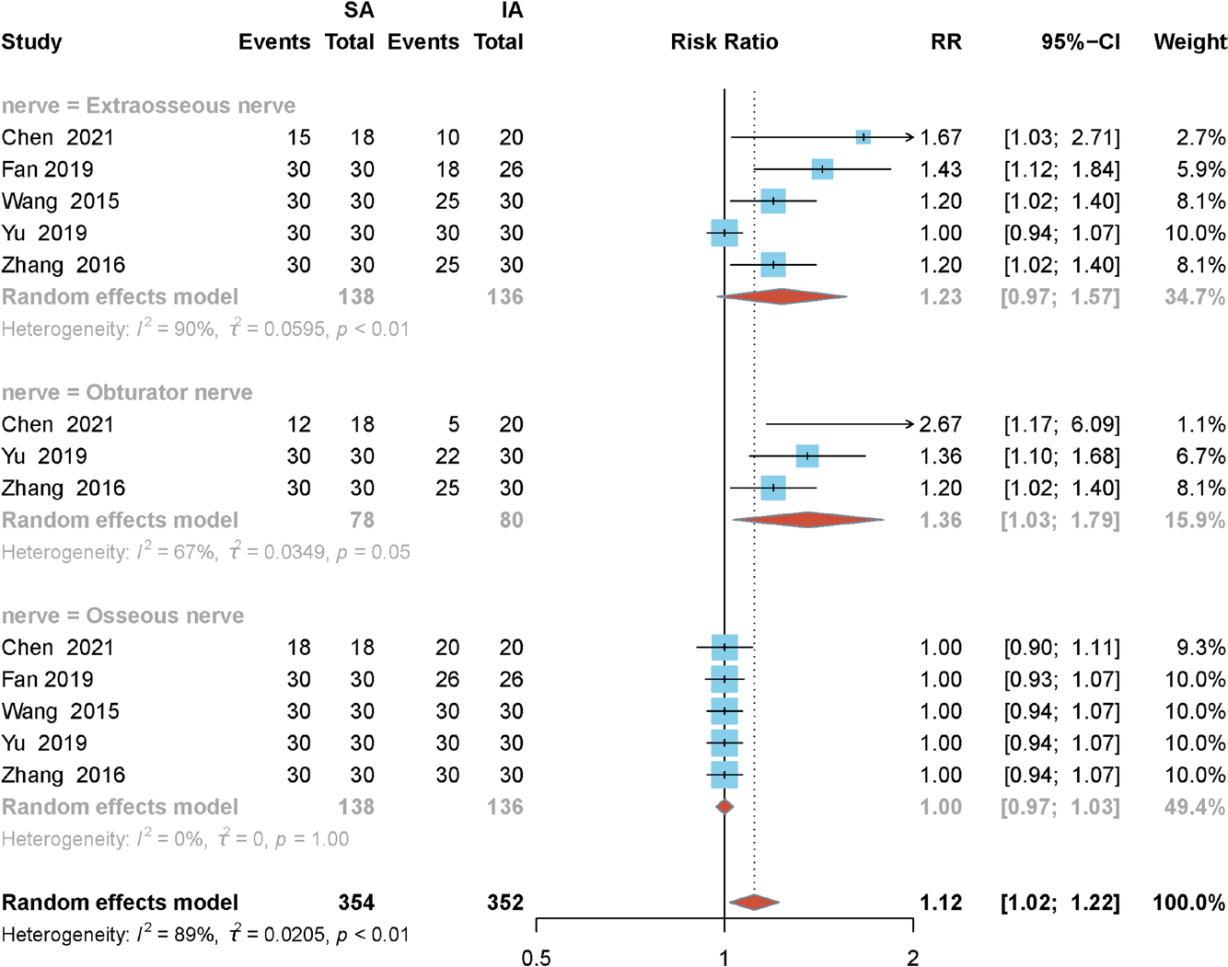
Forest plot of nerve block rates for SA and IA puncture pathway approaches for hip arthroplasty. SA, suprainguinal approach; IA, infrainguinal approach.

Meta-analysis of the effect of implementing SA and IA puncture routes on nerve block rates^[13,17,18,20,21]^ in the included literature was performed, which were divided into 3 groups according to the blocked nerves: the lateral femoral cutaneous nerve group, the femoral nerve group, and obturator nerve group.^[13,17,19]^ The heterogeneity test found that I^2^ = 90% in the lateral femoral cutaneous nerve group, I^2^ = 67% in the obturator nerve group, and I^2^ = 0% in the femoral nerve group. There was heterogeneity between the lateral femoral cutaneous nerve group and the obturator nerve group included in the literature, which may also be due to the difference in technique of the different puncture operators in the study. There was no significant difference between SA and IA puncture routes for block rates of the femoral nerve and obturator nerve groups [RR = 1.0, 95% confidence interval [CI] (1.02, 1.22) and RR = 1.36, 95% CI (1.03, 1.79), respectively]. However, there was a difference in the rate of the lateral femoral cutaneous nerve block between the SA and IA puncture paths, and the SA puncture path had a higher rate of the lateral femoral cutaneous nerve block than the IA puncture path [RR = 1.23, 95% CI (0.97, 1.57), *P* < .01], as detailed in Figure 3.

### 3.4. Effect of SA and IA puncture routes for FICB puncture time

**Figure 4.**
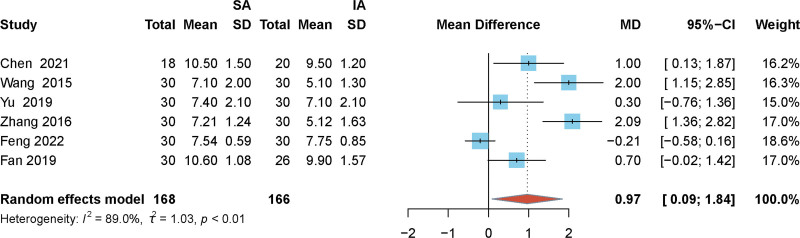
Forest plot of puncture times for hip arthroplasty SA and IA puncture pathway modalities. SA, suprainguinal approach; IA, infrainguinal approach.

The puncture time of SA and IA for FICB^[13,17–21]^ was compared in the included literature (including 168 patients in the SA group and 166 patients in IA group). The heterogeneity test results (I^2^ = 89% > 50%) showed that there was heterogeneity among the studies, and analysis of the heterogeneity found that this might have been due to differences in the technical proficiency of the practitioners. Meta-analysis of the results showed that the procedure time of patients undergoing SA was longer than that of IA (MD = 0.97, 95% CI: 0.09, 1.84, *P* < .01, Fig. 4).

### 3.5. Effect of SA and IA puncture routes on VAS scores during activity

**Figure 5.**
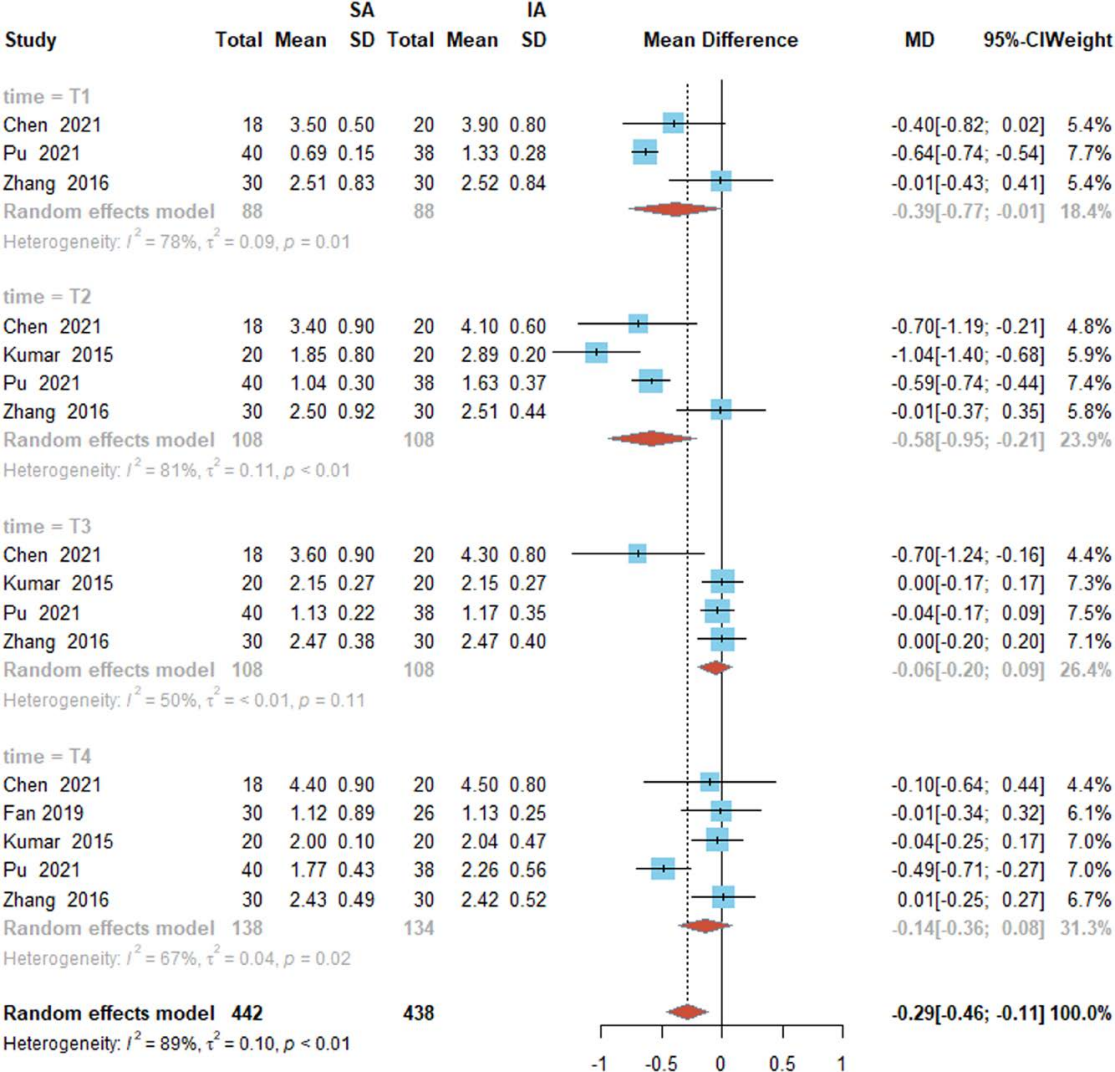
Forest plot of VAS scores for SA and IA in hip arthroplasty. SA, suprainguinal approach; IA, infrainguinal approach.

Meta-analysis of VAS scores^[13,17,18,20,21]^ for SA and IA were analyzed. Subjects were divided into 4 groups based on the length of time after the activity. The T1 group was 3 to 6 hours after the activity, T2 was 8 hours after the activity, T3 was 12 hours after the activity, and T4 was 24 hours after the activity. The heterogeneity test found that the I^2^ of the T1 to 4 groups were 78%, 81%, 50%, and 67%, respectively. Due to the certain degree of heterogeneity among the included studies in each group, the random-effects model was used for analysis. Meta combined effect size results for group T1 [MD = −0.39, 95% CI (−0.77, −0.01), *P* = .01], group T2 [MD = −0.58, 95% CI (−0.95, −0.21), *P* < .01], group T3 [MD = −0.06, 95% CI (−0.20, 0.09), *P* = .11]. T4 group [MD = −0.14, 95% CI (−0.36, 0.08), *P* < .01], respectively. It was found that patients’ VAS scores during activity were lower in the T1, T2, and T4 groups than in the IA puncture path group for the SA puncture path, indicating that the SA puncture path could reduce patients’ pain during postoperative activity, and the difference was statistically different. However, there was no statistically significant difference between the 2 groups of patients in the T3 group, as detailed in Figure 5.

### 3.6. Impact of SA and IA puncture routes on patients’ postoperative adverse reaction rates

**Figure 6.**
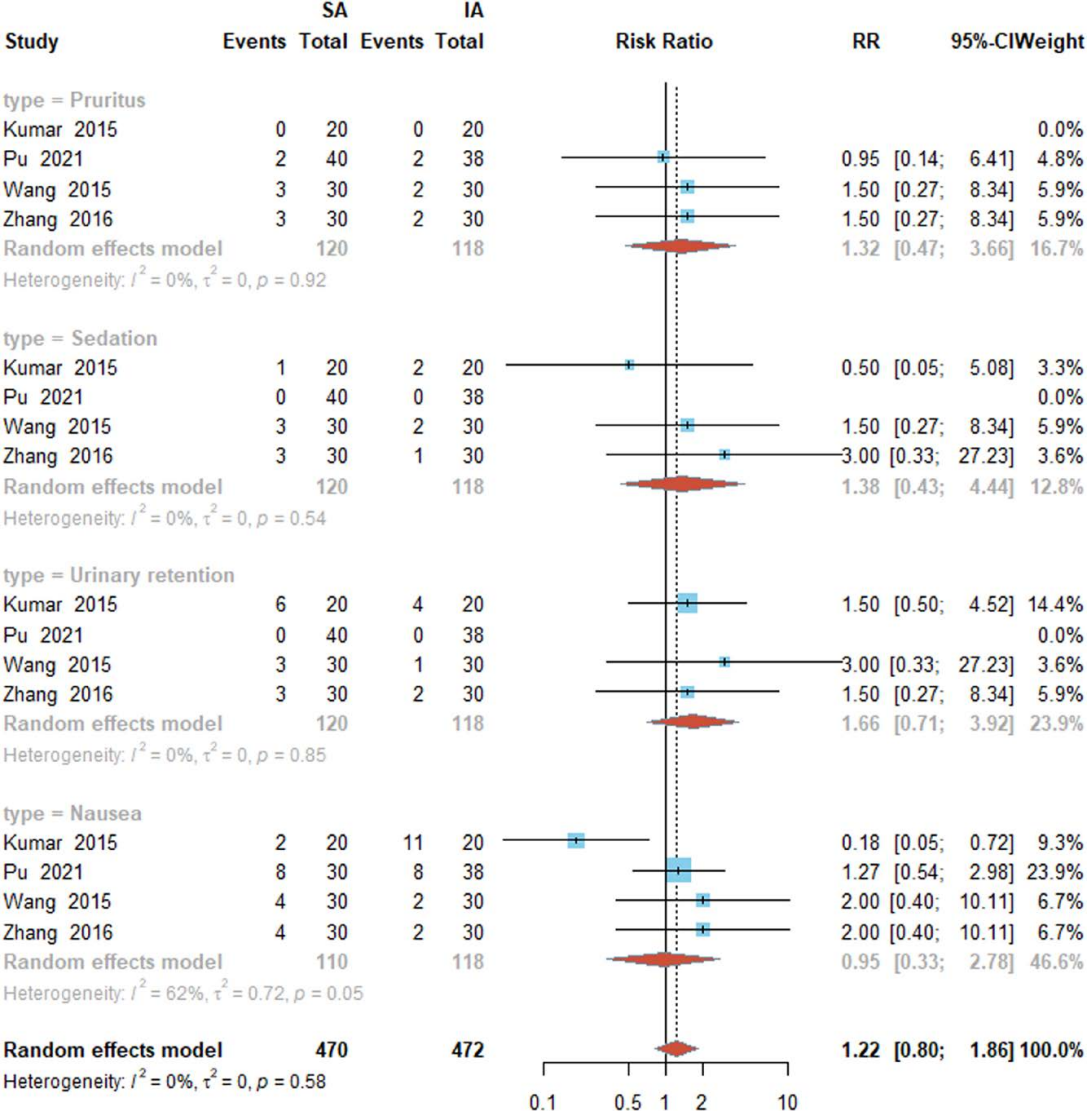
Forest plot of the adverse reaction rate for SA and IA in hip arthroplasty. SA, suprainguinal approach; IA, infrainguinal approach.

Meta-analysis of the postoperative adverse reaction rates^[15–17,21]^ of patients for SA and IA was conducted. Based on the postoperative adverse reactions of patients, subjects were divided into the pruritus, sedation, urinary retention, and nausea and vomiting groups. The I^2^ of the 4 groups were 0%, 0%, 0%, and 62%, respectively. There was low heterogeneity in the pruritus, sedation, and urinary retention groups, but there was moderate heterogeneity in the nausea and vomiting group. Therefore, the random-effects model was still used for meta-analysis. Meta-analysis of the results showed that the itching group [RR = −1.32, 95% CI (0.47, 3.66), *P* = .92], the sedation group [RR = 1.38, 95% CI (0.43, 4.44), *P* ≤ .54], and the urinary retention group [RR = 1.66, 95% CI (0.71, 3.92), *P* = .85], the nausea and vomiting group [RR = 0.95, 95% CI (0.33, 2.78), *P* = .58], and it was found that the 2 puncture routes of SA and IA had no effect on the incidence of postoperative adverse reactions in patients undergoing hip arthroplasty, and the difference was not statistically significant, as shown in Figure 6.

## 4. Discussion

Postoperative pain management after hip arthroplasty is the main problem affecting a patient’s postoperative recovery. Several strategies to reduce postoperative pain have been reported, including oral and muscle relaxation, local surgical site block, nerve block, intravenous controlled analgesia, and epidural controlled analgesia, among others.^[22]^ Among them, FICB, as an important method of postoperative analgesia after hip arthroplasty, the difference in the puncture pathway on the patient’s postoperative outcome as well as the difference in the complication rate has already been reported in several papers,^[13,15,16]^ and in this paper, by comprehensively analyzing the existing literature, we found that the SA puncture pathway was significantly superior to the IA pathway in terms of pain control in the early postoperative period (within 3–6 and 8 hours), as evidenced by lower pain scores and success rate of the lateral femoral cutaneous nerve block.^[17,20,21]^ However, the 2 pathways did not show a significant difference in VAS scores and incidence of adverse events at 12 hours postoperatively, which is in line with the findings of Kumar et al,^[15]^ who reported that the SA pathway improves pain in the early postoperative period, but that the effect is not statistically different over a longer time period. In contrast, the study by Chen et al^[13]^ found that the SA puncture route reduced patients’ postoperative pain scores at 8 and 12 hours postoperatively and did not make a difference in postoperative pain scores at 24 hours postoperatively. In the study by Pu et al^[16]^ significantly reduced patients’ postoperative pain differences at 4 hours, 8 hours, 12 hours and 24 hours postoperatively. This series of findings highlights the potential value of the SA puncture pathway in early pain control when developing analgesic regimens after hip arthroplasty,^[13,19]^ particularly for those patients who require rapid early rehabilitation.

Peripheral block anesthesia offers targeted pain relief with fewer systemic side effects, often reducing the need for opioids and allowing for quicker recovery and improved patient satisfaction. It is essential to compare the efficacy and safety of different peripheral anesthetic blocks in surgeries. For instance, in geriatric hip fracture surgery, the pre-operative Pericapsular Nerve Group block has proven to be more effective than the femoral nerve block in reducing pain during spinal anesthesia positioning. The Pericapsular Nerve Group block provides targeted pain relief to the hip joint without causing motor blockade, making it safer for elderly patients and potentially reducing the need for postoperative opioids.^[23]^ In hallux valgus percutaneous surgery, there was no significant difference in effectiveness between the femoral-sciatic nerve block and ankle block regional anesthesia.^[24]^

According to research by Fantoni I et al, patients with hip osteoarthritis show higher levels of Collagen type I and lower levels of Collagen type III and hyaluronan in the fascia lata compared to healthy individuals, suggesting a link between fascial pathology and the development of osteoarthritis symptoms.^[25]^ These collagen and hyaluronan changes can influence the fascial gliding, impairing muscle and joint biomechanics, potentially leading to pain and function loss. Fascial alteration is related to the development of myofascial pain, for richly innervated by small nerve fibers that may influence pain perception.^[26]^ Several studies have demonstrated the superiority of suprainguinal FICB in providing a greater and more consistent cephalic spread of local anesthetic, leading to better lumbar plexus blockade and improved analgesic effects compared to infrainguinal FICB,^[27,28]^ aligning with our results in early postoperative period.

However, these advantages did not emerge over a longer postoperative period, that is, the difference between the 2 pathways was not significant in terms of VAS scores and incidence of adverse events at 12 hours. This finding is consistent with previous findings, suggesting a specific advantage of the SA pathway for early pain control and a more limited impact on long-term pain management. Consistent with the findings of Kumar et al^[15]^ and other related studies, which further emphasize the potential benefits of the SA pathway in early postoperative pain control, its benefits in the longer postoperative period have failed to be clearly demonstrated, as reflected in the incidence of adverse events. This result suggests a specific advantage of the SA pathway in early pain control, while its impact on long-term pain management is relatively limited.

Although this study provides valuable insights, its limitations cannot be ignored. Firstly, the amount of literature included was limited and there may have been methodological differences. For example, technical proficiency, pain scoring methods, and pain management protocols may have been inconsistent across studies, all of which may have had an impact on the results. Secondly, some studies failed to implement a double-blind design, which may increase the risk of bias. Therefore, future studies need to adopt a more rigorous design and include a wider range of literature to further validate our findings.

## 5. Conclusions

In summary, the results of this meta-analysis support the use of the SA puncture pathway for FICB to optimize early pain management after hip arthroplasty, particularly in significantly reducing pain scores and increasing the rate of the lateral femoral cutaneous nerve block at 3 to 6 and 8 hours postoperatively. This finding provides important guidance to clinicians to help them make more informed choices when developing analgesic regimens after hip arthroplasty. Future studies should focus on validating the efficacy and safety of different FICB puncture pathways in postoperative analgesic management after hip arthroplasty through larger, multicentre RCTs. In addition, exploring individualized pain management strategies, such as selecting puncture pathways based on patient-specific needs and rehabilitation goals, will be important for improving postoperative pain control and patient satisfaction.

## Author contributions

**Conceptualization:** Zhi Yang.

**Data curation:** Zhi Yang, Wang Xu.

**Formal analysis:** Zhi Yang, Wang Xu.

**Funding acquisition:** Zhi Yang.

**Project administration:** Wang Xu.

**Writing – original draft:** Zhi Yang, Wang Xu.

**Writing – review & editing:** Zhi Yang, Wang Xu, Shan Xu.

## References

[1] Scurrah A Shiner CT Stevens JA Faux SG. Regional nerve blockade for early analgesic management of elderly patients with hip fracture - a narrative review. Anaesthesia. 2018;73:769–83. 29278266 10.1111/anae.14178

[2] Roberts KC Brox WT Jevsevar DS Sevarino K. Management of hip fractures in the elderly. J Am Acad Orthop Surg. 2015;23:131–7. 25624365 10.5435/JAAOS-D-14-00432

[3] Berend KR Lombardi AV JR Berend ME Adams JB Morris MJ. The outpatient total hip arthroplasty: a paradigm change. Bone Joint J. 2018;100-B(1 Supple A):31–5. 29292337 10.1302/0301-620X.100B1.BJJ-2017-0514.R1 PMC6424444

[4] Mamlin LA Melfi CA Parchman ML. Management of osteoarthritis of the knee by primary care physicians. Arch Fam Med. 1998;7:563–7. 9821832 10.1001/archfami.7.6.563

[5] Ceolin C Bano G Biz C. Functional autonomy and 12-month mortality in older adults with proximal femoral fractures in an orthogeriatric setting: risk factors and gender differences. Aging Clin Exp Res. 2023;35:1063–71. 36892795 10.1007/s40520-023-02378-y

[6] Bano G Dianin M Biz C. Efficacy of an interdisciplinary pathway in a first level trauma center orthopaedic unit: a prospective study of a cohort of elderly patients with hip fractures. Arch Gerontol Geriatr. 2020;86:103957. 31698279 10.1016/j.archger.2019.103957

[7] Liao KM Lu HY. A national analysis of complications following total hip replacement in patients with chronic obstructive pulmonary disease. Medicine (Baltimore). 2016;95:e3182. 27015210 10.1097/MD.0000000000003182 PMC4998405

[8] Gola W Bialka S Owczarek AJ Misiolek H. Effectiveness of fascia iliaca compartment block after elective total hip replacement: a prospective, randomized, controlled study. Int J Environ Res Public Health. 2021;18:4891. 34064427 10.3390/ijerph18094891 PMC8124308

[9] Liu X Hu X Li R Zhang Y. Combination of post-fascia iliaca compartment block and dexmedetomidine in pain and inflammation control after total hip arthroplasty for elder patients: a randomized control study. J Orthop Surg Res. 2020;15:42. 32041613 10.1186/s13018-020-1562-6 PMC7011216

[10] Swenson JD Davis JJ Stream JO Crim JR Burks RT Greis PE. Local anesthetic injection deep to the fascia iliaca at the level of the inguinal ligament: the pattern of distribution and effects on the obturator nerve. J Clin Anesth. 2015;27:652–7. 26277873 10.1016/j.jclinane.2015.07.001

[11] Zhang XY Ma JB. The efficacy of fascia iliaca compartment block for pain control after total hip arthroplasty: a meta-analysis. J Orthop Surg Res. 2019;14:33. 30683117 10.1186/s13018-018-1053-1 PMC6347785

[12] Eyi YE Arziman I Kaldirim U Tuncer SK. Fascia iliaca compartment block in the reduction of dislocation of total hip arthroplasty. Am J Emerg Med. 2014;32:1139. 10.1016/j.ajem.2014.06.007 25027195

[13] Chen L Shen Y Liu S Cao Y Zhu Z. Ultrasound-guided supra-inguinal fascia Iliaca compartment block for older adults admitted to the emergency department with hip fracture: a randomized controlled, double-blind clinical trial. BMC Geriatr. 2021;21:669. 34852764 10.1186/s12877-021-02646-4 PMC8638559

[14] Bullock WM Yalamuri SM Gregory SH Auyong DB Grant SA. Ultrasound-guided suprainguinal fascia iliaca technique provides benefit as an analgesic adjunct for patients undergoing total hip arthroplasty. J Ultrasound Med. 2017;36:433–8. 27943417 10.7863/ultra.16.03012

[15] Kumar K Pandey RK Bhalla AP. Comparison of conventional infrainguinal versus modified proximal suprainguinal approach of Fascia Iliaca compartment block for postoperative analgesia in total hip arthroplasty. a prospective randomized study. Acta Anaesthesiol Belg. 2015;66:95–100. 26767235

[16] Pu M Xu J Xu X Xiang J Xie X. Comparative analysis of analgesic effect of iliac fascial block with vertical and horizontal inguinal approach for total hip arthroplasty. Am J Transl Res. 2021;13:9593–9. 34540083 PMC8430063

[17] Wang N Li M Wei Y Guo X. A comparison of two approaches to ultrasound-guided fascia iliaca compartment block for analgesia after total hip arthroplasty. Zhonghua yi xue za zhi. 2015;95:2277–81. 26710951

[18] Jian Y Chunhua Z Yajun J. Comparison of different approaches to fascia iliaca compartment block for postoperative analgesia in elderly patients undergoing total hip arthroplasty. Chin J Anesthesiol. 2019;39:1224–7.

[19] Feng T Zhao J Wang J Sun X Jia T Li F. Anesthetic effect of the fascia iliaca compartment block with different approaches on total hip arthroplasty and its effect on postoperative cognitive dysfunction and inflammation. Front surg. 2022;9:898243. 35599808 10.3389/fsurg.2022.898243 PMC9114884

[20] Yaling F Gang Z Xu C. Application of ultrasound-guided continuous fascia iliaca compartment block through different approaches in total hip arthroplasty. J Clin Anesthesiol. 2019;35:247–52.

[21] Li Z Quan L Ling S. Comparison of analgesic effects of different iliac fascia block under the guidance of ultrasound in the patients after hip replacement. Chin J Front Med Sci (Electronic Version). 2016;8:46–9.

[22] Kehlet H Dahl JB. Anaesthesia, surgery, and challenges in postoperative recovery. Lancet (London, England). 2003;362:1921–8. 14667752 10.1016/S0140-6736(03)14966-5

[23] Erten E Kara U Simsek F. Comparison of pericapsular nerve group block and femoral nerve block in spinal anesthesia position analgesia for proximal femoral fractures in geriatric patients: a randomized clinical trial. Ulus Travma Acil Cerrahi Derg. 2023;29:1368–75. 38073453 10.14744/tjtes.2023.33389 PMC10767289

[24] Biz C De Iudicibus G Belluzzi E. Prevalence of chronic pain syndrome in patients who have undergone hallux valgus percutaneous surgery: a comparison of sciatic-femoral and ankle regional ultrasound-guided nerve blocks. BMC Musculoskelet Disord. 2021;22:1043. 34911525 10.1186/s12891-021-04911-4 PMC8675526

[25] Fantoni I Biz C Fan C. Fascia lata alterations in hip osteoarthritis: an observational cross-sectional study. Life (Basel). 2021;11:1136. 34833012 10.3390/life11111136 PMC8625990

[26] Fede C Porzionato A Petrelli L. Fascia and soft tissues innervation in the human hip and their possible role in post-surgical pain. J Orthop Res. 2020;38:1646–54. 32181900 10.1002/jor.24665

[27] Desmet M Balocco AL Van Belleghem V. Fascia iliaca compartment blocks: different techniques and review of the literature. Best Pract Res Clin Anaesthesiol. 2019;33:57–66. 31272654 10.1016/j.bpa.2019.03.004

[28] Bansal K Sharma N Singh MR Sharma A Roy R Sethi S. Comparison of suprainguinal approach with infrainguinal approach of fascia iliaca compartment block for postoperative analgesia. Indian J Anaesth. 2022;66(Suppl 6):S294–9. 36425915 10.4103/ija.ija_823_21 PMC9680722

